# Responsiveness of Urinary and Plasma Alkylresorcinol Metabolites to Rye Intake in Finnish Women

**DOI:** 10.3390/cancers2020513

**Published:** 2010-04-14

**Authors:** Mylène Aubertin-Leheudre, Anja Koskela, Adile Samaletdin, Herman Adlercreutz

**Affiliations:** Folkhälsan Research Center, Institute for Preventive Medicine, Nutrition and Cancer, and Division of Clinical Chemistry, University of Helsinki, Haartmaninkatu 8, P.O. Box 63, FI-00014, Finland; E-Mails: anja.koskela@helsinki.fi (A.K.); adile.samaletdin@helsinki.fi (A.S.); herman.adlercreutz@helsinki.fi (H.A.)

**Keywords:** whole-grain cereals, rye, alkylresorcinol metabolites, responsiveness

## Abstract

Alkylresorcinols [ARs] have been proposed for use as biomarkers of whole-grain intake. The aim here was to examine the responsiveness of AR metabolites to rye intake. Sixty women were divided into three groups according to their rye consumption. We observed significant differences between groups in plasma 3-[3,5-dihydroxyphenyl]-1-propanoic acid [DHPPA] and in urinary DHPPA and 3,5-dihydroxybenzoic acid [DHBA]. In addition, these AR metabolites increased proportionally to rye fiber intake. We conclude that these ARs metabolites are accurate and useful biomarkers of rye fiber intake. Further studies are needed to confirm our results in larger and different populations.

## 1. Introduction

Epidemiologic evidence for beneficial effects of whole**-**grain consumption on risk of several chronic diseases, including cardiovascular disease, diabetes, obesity, and certain types of cancer, has emerged [1,2]. These effects are caused by several components of the dietary fiber complex [3]. These studies are generally based on self-reported whole-grain intake. However, assessment of self-reported whole-grain intake could lead to measurement errors [4]. Furthermore, different definitions of whole grain have been used in different studies and countries [5]. Because of the poor accuracy in measuring intake of foods and nutrients [6] and the inherent weaknesses of food frequency questionnaires [3,4] in nutritional epidemiologic data, specific biomarkers for whole-grain cereal fiber intake could add critical support to epidemiological results and would overcome some serious drawbacks. Alkylresorcinols [ARs], amphilic 1,3-dihydroxy-5-alkylbenzene homologs with odd-numbered alkyl side-chains from C17:0 to C25:0 [7], are found in abundance in the fiber layers of rye and wheat grains, but are absent or limited in germ or endosperm layers and in most other foods [8]. AR intake can be followed by determination of their metabolites (3,5-dihydroxybenzoic acid: DHBA and 3-[3,5-dihydroxyphenyl]-1-propanoic acid: DHPPA) in plasma and in urine [9,10,11]. Ross *et al.* [12] showed that 45–71% of ingested ARs are absorbed. ARs have been proposed to function as biomarkers for whole-grain intake due to these properties [8,13]. Because of the specific occurrence of ARs in the fiber complex of whole-grain wheat and rye, intact plasma ARs and urinary AR metabolites have been proposed to be better biomarkers than enterolactone for the consumption of fiber-rich cereals in Nordic [14,15,16] and American populations [11]. However, the half-life of intact plasma ARs is around 5 h, which is a short or medium half-life [17]. AR metabolites are possibly formed via β-oxidation of intact plasma ARs [8]. The responsiveness of intact ARs and urinary metabolites in an interventional rye bread study was previously demonstrated to be related to the amount of rye ingested by subjects, during the protocol [18]. However, we showed that AR metabolites, and more specifically AR metabolites in plasma, correlate more strongly with cereal fiber intake than do intact plasma ARs [14,19]. In addition, our preliminary analyses indicated, that the half-life of plasma AR metabolites is longer than 5 h (~10–15 h).

AR metabolites seem to be more accurate biomarkers than intact plasma ARs of whole-grain rye and wheat intake [14,15,19]. To confirm that urinary and plasma AR metabolites can be used as sensitive and specific biomarkers, evaluation of the response of these metabolites to rye intake is important. Thus, our objective was to examine the responsiveness of urinary and plasma AR metabolites to rye intake in Finnish women.

## 2. Results and Discussion

We observed no significant difference between groups for age, weight, BMI, age at menopause, age at menarche, smoking status, physical status, age at first pregnancy, and number of children (Table 1). No significant differences between groups were detected for total energy intake, total fat intake, saturated fatty-acid (SFA), mono-unsaturated fatty-acid (MUFA), and poly-unsaturated fatty-acid (PUFA) all of which are potential confounders during AR absorption (Table 1; [23]). We found significant differences between groups for rye and cereal fiber intakes (Table 1). These results were expected since groups were divided based on the level of rye intake. No difference between groups was observed for wheat intake or total fiber intake (Table 1). Finally, significant differences emerged between groups for plasma DHPPA, urinary DHPPA, urinary DHBA, and sum of urinary AR metabolites, but not for plasma DHBA or sum of plasma AR metabolites (Table 1). 

The high rye intake group, compared with the medium and low rye intake groups, had a greater urinary DHPPA (p = 0.026 and p = 0.001, respectively), urinary DHBA (p = 0.042 and p = 0.001, respectively), and plasma DHPPA (p = 0.034 and p = 0.034, respectively). 

**Table 1:** Descriptive characteristics of the groups.

Variable	Low rye intake (n = 20)	Medium rye intake (n = 20)	High rye intake (n = 20)	p-values
Age (years)	47 ± 13	45 ± 15	49 ± 15	0.598
Weight (kg)	60 ± 11	61 ± 8	63 ± 6	0.648
Body mass index (kg/m²)	22 ± 3	23 ± 3	23 ± 3	0.549
Age at menopause (years)	50 ± 3	50 ± 1	49 ± 4	0.768
Age at menarche (years)	13 ± 1	13 ± 1	13 ± 1	0.576
Smoker (%)	25	5	10	0.153*
Physically active (%)	55	50	50	0.935*
Age at first pregnancy (years)	26 ± 3	27 ± 5	25 ± 5	0.521
Number of children	1.9 ± 1.5	1.1 ± 1.3	1.5 ± 1.5	0.183
P-DHBA (nmol/L)P-DHPPA (nmol/L)	85 ± 5676 ± 37	97 ± 6989 ± 53	102 ± 45110 ± 43^∆†^	0.333**0.043**
U-DHBA (μmol/24h)	21 ± 8	26 ± 12	32 ± 9^∆†^	0.005**
U-DHPPA (μmol/24h)	32 ± 15	40 ± 26	48 ± 13^∆†^	0.001**
Sum P-AR metabolites (nmol/L)	157 ± 87	187 ± 121	210 ± 87	0.185
Sum U-AR metabolites (μmol/24h)	52 ± 23	66 ± 38	79 ± 22^∆†^	0.003**
Rye intake (g/d)	23 ± 9^†^	44 ± 4^∆^	68 ± 18^∆^	0.000
Wheat intake (g/d)	79 ± 30	79 ± 20	71 ± 36	0.615
Cereal fiber intake (g/d)	7.8 ± 2.3^†^	9.4 ± 1.6^∆^	11.9 ± 2.6^∆†^	0.000
Total fiber intake (g/d)	16 ± 7	19 ± 5	20 ± 5	0.156
Total kilocalorie intake (kcal/d)	1774 ± 281	1866 ± 285	1832 ± 410	0.676
Total fat intake (g/d)	76 ± 15	76 ± 15	72 ± 18	0.682
SFA (g/d)	36 ± 11	39 ± 9	38 ± 11	0.715
MUFA (g/d)	25 ± 5	24 ± 6	22 ± 6	0.293
PUFA (g/d)	10 ± 3	10 ± 4	9 ± 4	0.825

Means ± SD. p < 0.05 = significantly different.* p-value obtained by Chi-square test. ** p-value obtained with log variables. ^∆^ p < 0.05; significantly different from low rye intake. ^†^ p < 0.05; significantly different from medium rye intake. Physically active = physical activity > 3 h/week; P = plasma; U = Urine; DHBA = 3,5-dihydroxybenzoic acid; DHPPA = 3-[3,5-dihydroxyphenyl]-1-propanoic acid; SFA = saturated fatty acid; MUFA = mono-unsaturated fatty acid; PUFA = poly unsaturated fatty acid.

**Figure 1:**
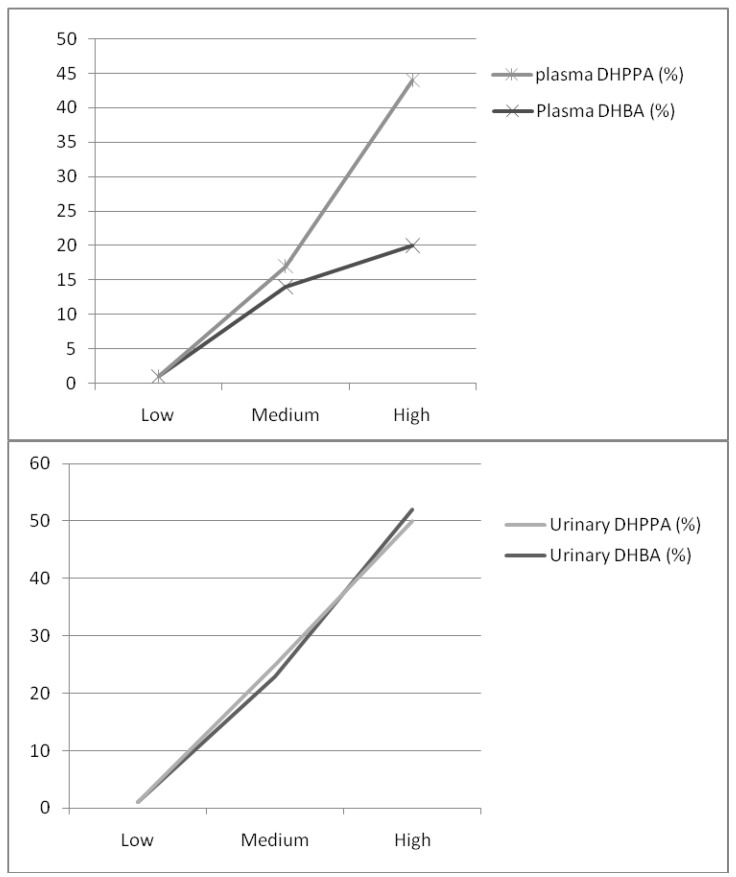
Responsiveness (%) of urinary and plasma alkylresorcinol metabolites between medium or high rye intake group and low rye intake group (reference).

Urinary DHBA correlated significantly and more strongly than plasma DHBA with rye intake (r = 0.524 *vs.* r = 0.299) and fiber intake (r = 0.443 *vs.* r = 0.331), Urinary DHPPA and plasma DHPPA correlated significantly with rye intake (r = 0.438 *vs.* r = 0.397) and total fiber intake (r = 0.390 *vs.* r = 0.366), even after adjustment for BMI and age (Table 2). We found that urinary AR metabolites and plasma DHPPA increased proportionally and significantly with the consumption of rye (Table 3; Figure 1). However, plasma DHBA did not significantly increase with greater rye intake (Table 3; Figure 1). In addition, the urinary AR metabolite ratio seemed relatively stable with different rye intakes but this was not the case with the plasma AR metabolite ratio (Table 3).

**Table 2:** Correlation between rye intake and alkylresorcinol metabolites with or without age and body mass index as covariables.

	Rye intake (g/d) with covariables	Rye intake (g/d) without covariables
P-DHBA (nmol/L)	0.299*	0.321*
P-DHPPA (nmol/L)	0.397**	0.385**
P-DHBA+DHPPA (nmol/L)	0.215	0.334*
U-DHBA (μmol/24h)	0.524***	0.516***
U-DHPPA (μmol/24h)	0.438***	0.444***
U-DHBA+DHPPA (μmol/24h)	0.365**	0.476***

* p < 0.05.** p < 0.01.*** p < 0.001. P = plasma; U = urine; DHBA = 3,5-dihydroxybenzoic acid; DHPPA = 3-[3,5-dihydroxyphenyl]-1-propanoic acid.

**Table 3:** Responsiveness of urinary and plasma alkylresorcinol metabolites to rye intake.

Rye intake groups	P-DHBA (nmol/L)	P-DHPPA (nmol/L)	P-DHBA/DHPPA (ratio)	U-DHBA (μmol/24h)	U-DHPPA (μmol/24h)	U-DHBA/DHPPA (ratio)
Low (reference)	1	1	1	1	1	1
Medium (+21 g/d)	+14%	+17%	+18%	23%	25%	+1%
High group (+45 g/d)	+20%	+44%^∆†^	+25%	52%^∆†^	50%^∆†^	−6%

P = plasma; U = urine; DHBA = 3,5-dihydroxybenozoic acid; DHPPA = 3-[3,5-dihydroxyphenyl]-1-propanoic acid. Number of subjects in each group = 20.∆ p < 0.05; significantly different from low rye intake.† p < 0.05; significantly different from medium rye intake.

We evaluated whether the amount of rye intake in a habitual diet would be related to the responsiveness of AR metabolites in urine and plasma similarly to in an interventional study [18]. Responsiveness is a prerequisite for a biomarker [24]. We allocated 60 women into three dose groups (reference dose 23 g/d *vs.* 44 g/d or 68 g/d of rye) and compared the response of AR metabolites relative to the amount of rye consumed. Compared with the reference dose, we observed that for an increase of 21 g/d and 45 g/d of rye, AR metabolites rose by 25% and 50%, respectively. Urinary AR metabolites were closely related to rye intake, with their levels increasing proportionally to the amount of rye consumed (Table 2, Table 3). However, plasma DHPPA responded much more strongly to rye intake in Finnish women. Our results confirmed those of Landberg *et al.* [18], who observed during their interventional study that 24-h urinary excretion of AR metabolites had a linear dose-response relation with plasma intact AR concentration. In addition, urinary metabolite excretion is known to correlate with plasma intact AR concentration. Finally, we found that responsiveness of DHPPA in plasma and urine is related to the amount of rye consumed. Interestingly, plasma DHPPA correlated more strongly with cereal fiber intake (r = 0.463; [19]) than plasma AR C21:0 (r = 0.416; [19]) or urinary DHBA (r = 0.372; [14]). In epidemiological studies, it is easier to obtain fasting blood samples than 24-h urine collections. Moreover, the half-life of plasma AR metabolites (mean ~12 h;) is longer than the half-life of intact plasma ARs (mean ~5 h, [17]). The method for analyzing plasma and urinary AR metabolites is more convenient, faster and requires less sample pre-treatment than the method for plasma intact ARs. 

Our study is the first to show that AR metabolites, more specifically AR metabolites in urine and plasma DHPPA, have a good responsiveness to wheat and rye whole-grain cereal intake [14,19]. Thus, our results are in line with those of Landberg *et al.* [18] and confirm that AR metabolites, and specifically AR metabolites in urine, are a promising biomarker for rye and wheat cereal whole-grain intake. To be considered a dietary biomarker, a marker must be validated in experimental and observational studies investigating the dose-response relationships [24]. However, further research is needed before concluding that ARs, and specifically AR metabolites, are strong dietary biomarkers [25,26]. 

ARs are absorbed in the upper gastrointestinal tract and incorporated into chylomicrons, which are distributed to the circulation through the lymphatic system [12,27], transported to the liver, and thereafter incorporated in HDL or VLDL or hypothetically through the enterohepatic circulation for urinary AR metabolites similarly to estrogen metabolites [23]. The AR level in erythrocytes is correlated with the plasma AR concentration in the fasting blood sample and also follows the changes in whole-grain intake [27,28]. Longer AR homologs are more easily incorporated into the membrane [29]. The metabolites are transported via the blood to the kidneys. DHPPA, which is a bigger molecule than DHBA, might not be taken up by the liver, and is probably directly excreted in bile and urine. This potential mechanism for both AR metabolites could explain the responsiveness difference in the AR metabolite ratio in plasma and urine. To support this hypothesis, a change has been shown in the elimination route after hepatic metabolism from urinary excretion to biliary excretion for tocopherols, which have some structural similarities to ARs [23,30]. Finally, we observed no difference between groups for the amount of fat intake. Different fat amounts could affect the intestinal absorption of such highly lipophilic micronutrients as ARs [23]. 

Our study has some limitations. It was carried out in Finnish women, who are known to consume a high daily amount of cereal fibers, rich in ARs, mainly from whole-grain rye and wheat [31]. Finland and Denmark have the highest intake of ARs of all European countries. The estimated average daily intake of ARs is around 11 mg/d in the United Kingdom, as compared with 40 mg/d in Finland [12,31]. In our subjects, the amount of rye corresponds, in general, with the amount of cereal fiber intake (for each additional 20 g/d of rye intake, we observed an additional 2 g/d of cereal fiber intake, see Table 1). Thus, confirming our results in countries where rye is not the first source of cereal fiber, e.g., the US or the United Kingdom, would be a good idea. It is recognized that there are some inherent weaknesses of food frequency questionnaires [3,4]. However, a three-day dietary record has been demonstrated to be valid for estimating dietary intakes in adults. In addition, we collected the dietary data in two occasions which reduced the risk of error. Finally, if some misidentification of groups has been done by the subjects this would be true in each group. Moreover, further studies using spot urine (e.g., morning fasting urine sample) are needed to confirm that the responsiveness of urinary AR metabolites is equivalent to a day collection; for epidemiological studies, collecting spot urine would be easier than a 24-h urine collection.

## 3. Experimental

### 3.1. Subjects

Sixty women living in the Helsinki area were recruited. They were divided into three groups according to their rye intake: (1) low of rye intake: 23 ± 9 g/d (n = 20); (2) medium rye intake: 44 ± 4 g/d (n = 20), and (3) high rye intake: 68 ± 18 g/d (n = 20). Subjects with a history of cancer or other major diseases or using oral contraceptives, hormone replacement therapy, or antibiotics were excluded. All subjects agreed to consume their habitual diet throughout the study. Age, weight, height, body mass index (BMI: kg/m²), age at menarche, type of diet, number of children, menopausal status, smoking status, and physical activity level were recorded by a questionnaire during the screening visit. All subjects gave their informed consent and were initially interviewed by a doctor who disclosed the study plan. The research protocol was approved by the Ethics Committee of Helsinki University Central Hospital. 

### 3.2. Data Collection

All subjects were studied for five days on two occasions with a six-month interval. The five-day food record was initiated two days before the three-day plasma and urine collections. The food records included at least one day on the weekend. Fasting blood samples were taken every morning on three consecutive days. After cooling to room temperature, blood samples were centrifuged and plasma was collected. Sodium azide (0.1%) and ascorbic acid (0.1%) were added, and samples were stored at 4 °C until the last collection day, when equal portions of each plasma sample were combined and stored at −20 °C until analyzed. The three-day urine was collected in plastic bottles containing 1 g of ascorbic acid per liter volume. Sodium azide (0.1%) was added, and the samples were stored at −20 °C until analysis. The premenopausal women collected the samples during the mid-follicular phase of their menstrual cycles (days 5–7).

### 3.3. Dietary Intake

All subjects agreed to maintain their habitual diet throughout the study. Each subject was provided with a letter balance and was instructed on how to complete the dietary records. A three-day dietary record has been demonstrated to be valid for estimating dietary intakes in adults without cognitive impairments [20]. Dietary analyses were completed by a nutritionist using the Southgate tables for cereal fiber data [21], but otherwise using data produced by the manufacturers of the breads and other cereal products. 

### 3.4. Urinary Alkylresorcinol Metabolites

The two urinary AR metabolites, DHPPA and DHBA, were analyzed by high-performance liquid chromatography (HPLC) with coulometric electrode array detection (CEAD; ESA Biosciences) as described by Koskela *et al.* [9]. Briefly, to 100 µL of urine, we added the internal standard syringic acid in 10 µL of methanol. The sample was hydrolyzed overnight at 37 °C with an equal volume (100 µL) of hydrolysis solution containing 0.1 mol/L Na-acetate buffer, pH 5, 0.2 U/mL β-glucuronidase, and 2 U/mL sulfatase. After incubation, a 50-µL aliquot (equal to 25 µL of urine) was taken and 50 µL of methanol and 650 µL of HPLC mobile phase were added to the sample and the sample was analyzed for the two AR metabolites by HPLC-CEAD. The intra-assay CV was for DHBA 5.6% and for DHPPA 7.7%. The inter-assay CV was for DHBA 10.9% and for DHPPA 10.4%. The method is considered accurate, specific, and reproducible [9].

### 3.5. Plasma Alkylresorcinol Metabolites

The two plasma AR metabolites, DHPPA and DHBA, were analyzed by HPLC-CEAD as described by Koskela *et al.* [22]. Briefly, to 100 µL of plasma, we added syringic acid as the internal standard. The enzymatic hydrolysis was carried out overnight at 37 °C. The sample was acidified to reach a pH of 3 and thereafter extracted three times with diethyl ether. The combined organic phases were evaporated to dryness. The sample was reconstituted in 50 µL of methanol, and 100 µL of HPLC mobile phase was added. The sample was filtered through a 0.2-µm Gelman GHP filter and analyzed with HPLC-CEAD [22]. The intra-assay CV was for DHBA 4.2% and for DHPPA 3.8%. The inter-assay CV was for DHBA 7.4% and for DHPPA 10.7%. The method is considered accurate, specific, and reproducible [22]. 

### 3.6. Statistical Analysis

Normality of distribution was determined using the Kurtosis test. Data were log-transformed if abnormally distributed. The three groups (low, medium, and high) were compared by ANOVA using Bonneferoni *post-hoc* for all variables. Furthermore, we examined potential differences in responsiveness of plasma and urinary AR metabolites using parametric tests. Pearson´s and Partial (with age and BMI as covariables) correlations between urinary or plasma AR metabolites and rye intake were performed. P-values of ≤0.05 were considered statistically significant. Analyses were performed using SPSS 15.0 software (Chicago, IL).

## 4. Conclusions

We conclude that plasma DHPPA, and urinary DHBA and DHPPA appear to be strong and accurate biomarkers of whole-grain cereal fiber intake. In evaluating ARs as a biomarker of whole-grain wheat and rye intake, we earlier have shown that ARs (intact, urinary, and plasma metabolites) correlate with cereal fiber intake [14,19,22]. In this study, we observed the responsiveness of AR metabolites in Finnish women. Thus, studies carried out by Landberg *et al.* under intervention conditions [15,17,18,32] and our studies [11,14,16,19,27,33] support the idea that AR metabolites appear to be good dietary biomarkers. Further research is needed to confirm our results in larger and non-Nordic European populations where rye is not the first cereal fiber source. Finally, the kinetics of AR metabolites must be investigated to elucidate the difference in responsiveness between plasma and urinary AR metabolites in humans.
